# Supplementary material for: Drosophila ovarian stem cell niche ageing involves coordinated changes in transcription and alternative splicing
Source: Nat Commun. 2025 Mar 16;16:2596. doi: 10.1038/s41467-025-57901-8 (PMC11911433; doi:10.1038/s41467-025-57901-8)
Supplement: Supplementary file 1 — Supplementary information [file 41467_2025_57901_MOESM1_ESM.pdf]

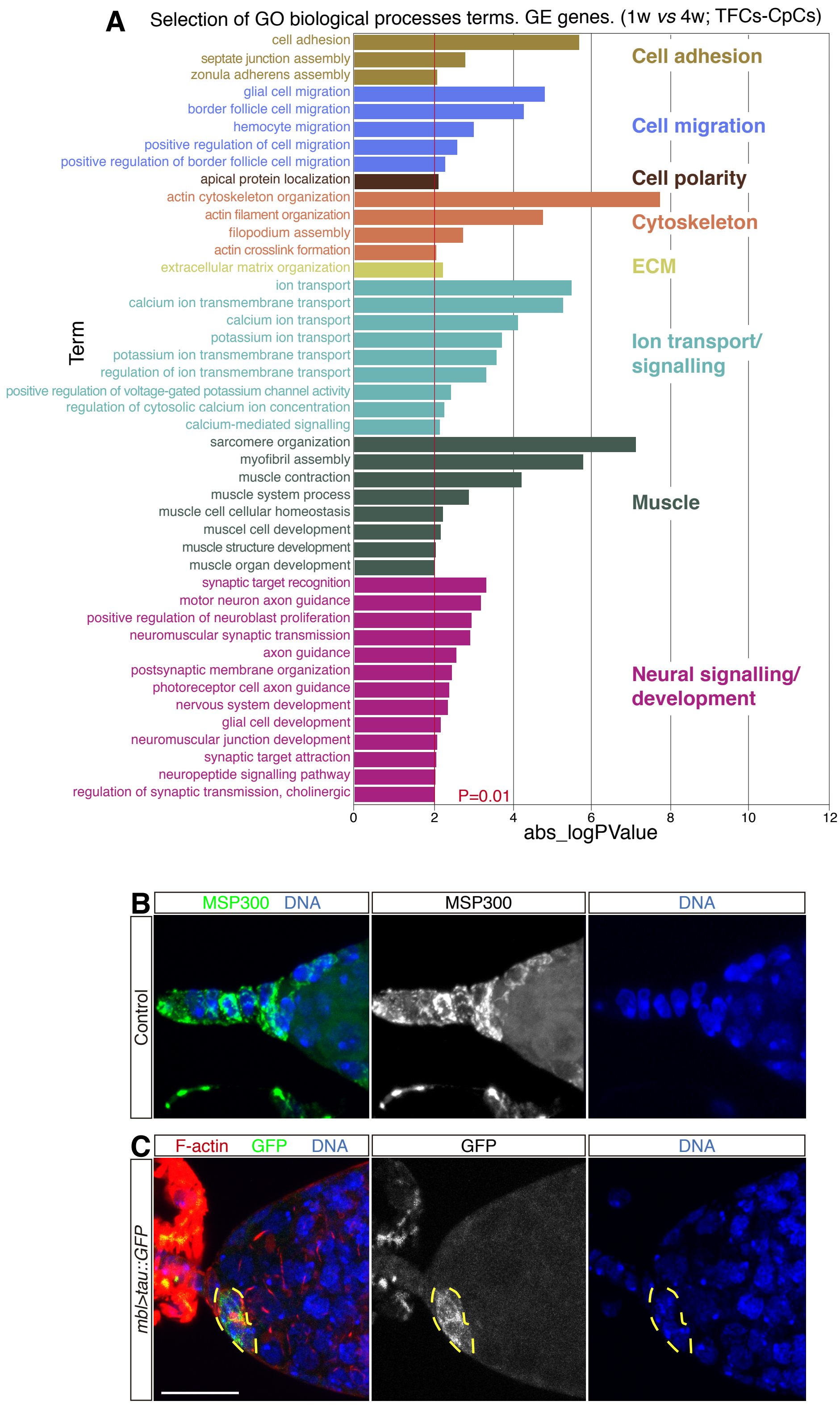

Fig. S1
Even-Ros et al.

Supplementary Figure 1: GO Biological Processes terms of genes expressed differentially during TFC-CpC ageing. Validation of genes identified in the GE analyses of TFCs-CpCs. (A) Representation of a selection of terms identified after the GO-biological processes terms analysis of genes with differential GE during TFC-CpC ageing. **(B)** Control germarium stained with anti-MSP300 (green) and the DNA dye Hoechst (blue). MSP300 is expressed in TFCs and CpCs. The images are a maximum projection of several z-sections across ~6 microns. **(C)** *mbl>tau::GFP* germarium stained with Rhodamine-Phalloidin (red), anti-GFP (green) and Hoechst (blue). This *mbl-Gal4* line is expressed in CpCs (yellow dotted line). Scale bar: 10µm. Related to Figure 1 and Supplementary Data 1.

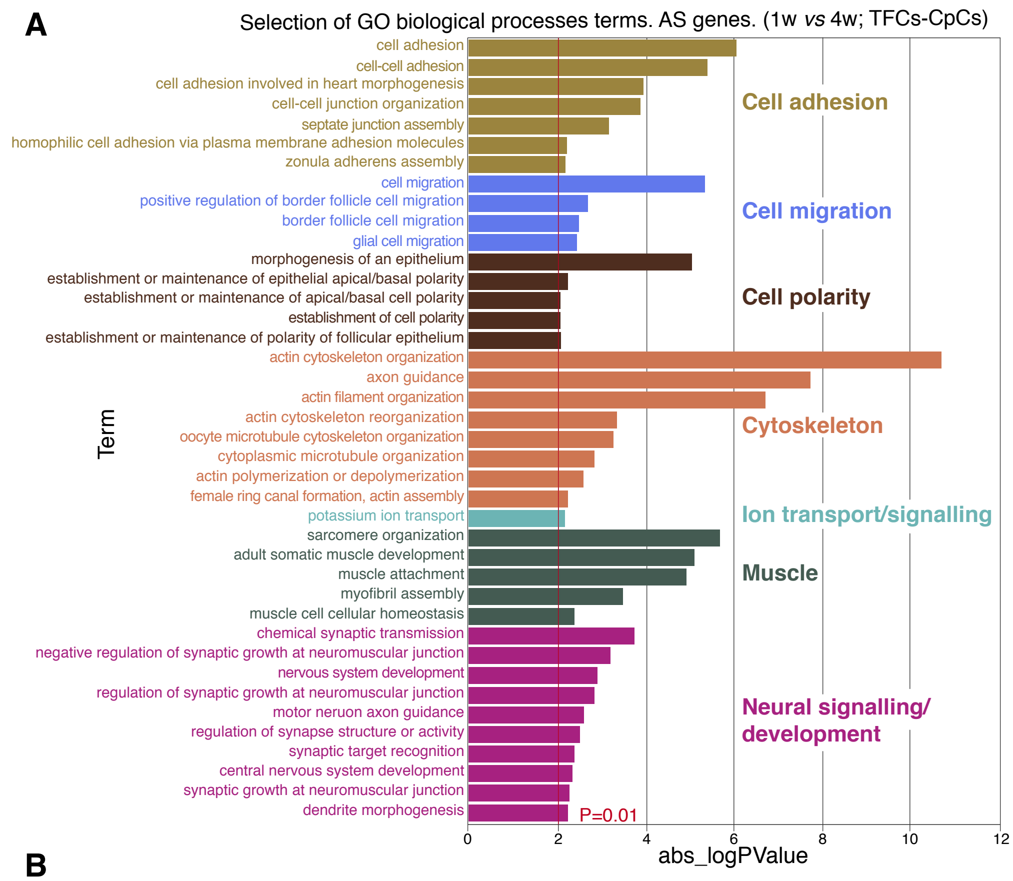

B

KEGG Term	P Value	Genes
Endocytosis	2.37E-04	Rab5, lqf, Src64B, PiP4K, step, Arp2, babo, Lerp, shi, Efa6, Nedd4, Amph, baz, Rip11, nuf, Asap, Snx6
Hippo signalling pathway	2.51E-04	sd, Gug, baz, sdt, Patj, scrib, dlg1, 14-3-3zeta, kibra, tws, zyx
Efferocytosis	7.67E-04	CaMKII, kuz, whd, Rab5, dor, hop, lt, CG32579, iPla2-VIA, sima, mbc
Inositol phosphate metabolism	0.019	norpA, fwd, CG3632, Pi3K68D, PiP4K, Plc21C, CG17896
Circadian rhythm	0.022	Pdp1, sgg, Atx2, cyc
Wnt signalling pathway	0.036	pan, gro, sgg, CaMKII, CanA-14F, nmo, Axn, Plc21C, CanA1, SkpA, norpA
Phosphatidylinositol signalling	0.069	norpA, fwd, CG3632, Pi3K68D, l(1)G0196, PiP4K, Plc21C
MAPK signalling pathway	0.078	Mef2, grk, vap, gro, Src64B, tor, msn, alph, 14-3-3zeta
FoxO signalling pathway	0.088	chico, foxo, lkb1, nmo, babo, CG11658, SNF4Agamma

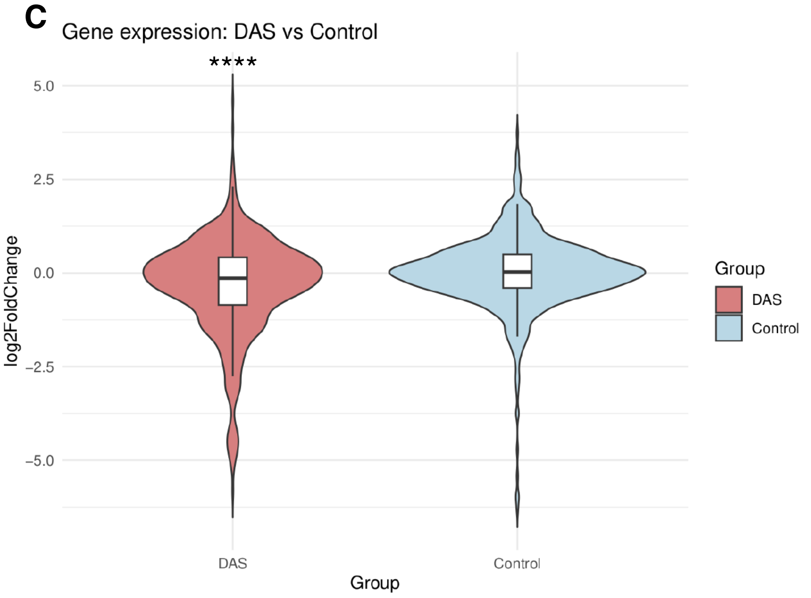

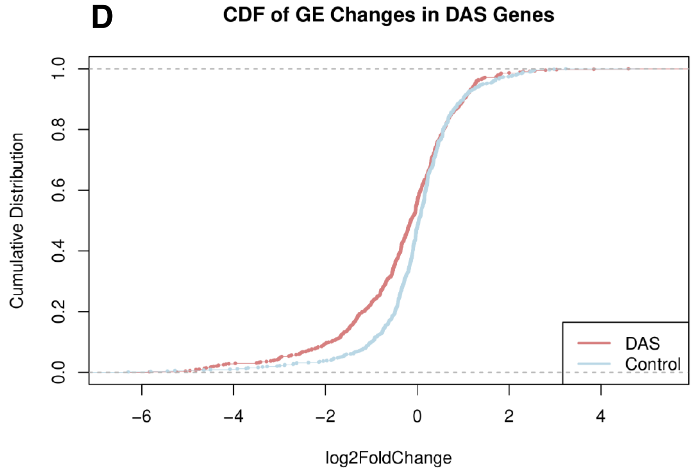

Fig. S2
Even-Ros et al.

Supplementary Figure 2: GO Biological Processes terms of genes showing AS changes during TFC-CpC ageing. Genes belonging to signalling pathways associated with alternatively spliced genes during ageing. (A) Representation of a selection of the terms identified after a GO-biological processes terms analysis of AS genes during TFC-CpC ageing. **(B)** List of all genes showing AS changes in TFCs-CpCs and that belong to any of the 9 KEGG pathways listed in Fig 2E. Gene names in red also present changes in GE. **(C)** Distribution of log2 fold change values of genes with differential AS (DAS) and control genes without. **(D)** Cumulative Distribution Function (CDF) of the genes in the DAS and Control categories. P value of Wilcoxon signed-rank test is shown (****: $P \leq 0.0001$). Related to Figure 2 and Supplementary Data 1.

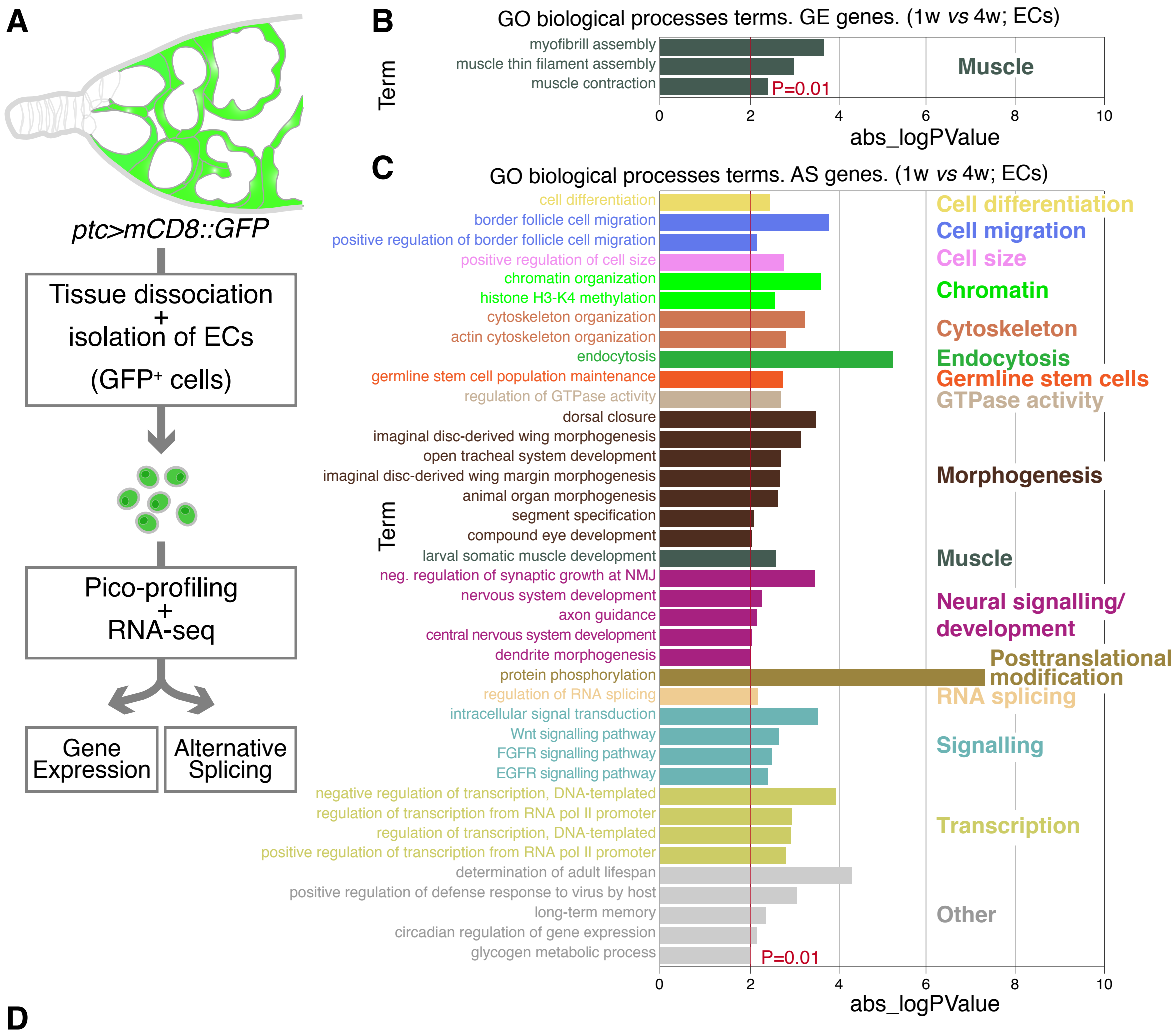

D

KEGG Term	P Value	Genes
Viral life cycle-HIV1	0.018	Cdk9, nej, Su(Tpl), Nelf-A, Fur1, Fur2
Efferocytosis	0.030	CamKII, csw, dor, p38a, rl, CG32579, sima, mbc
Polycomb repressive complex	0.037	nej, asx, wap, Su(z)12, Hcf, Kdm2, Sfmbt, ph-p
Autophagy	0.050	Atg17, CG3632, PI3K59F, Tak1, dor, Pten, Atg10, rl, sima, EndoB, Bruce
Inositol phosphate metabolism	0.059	fwd, CG3632, PI3K59F, PI4KIIalpha, Pten, IP3K1
Endocytosis	0.069	lqf, CG2224, babo, CenB1A, Nedd4, Pld, Amph, ArfGAP3, EndoB, CenG1A, Chc
Apoptosis	0.075	Dredd, eIF5, Tak1, tefu, Tab2, rl, Bruce
Glycerophospholipid metabolism	0.075	CG2201, Pect, CG2818, Pld, Lpin, sws, frj
FoxO signalling pathway	0.084	nej, babo, polo, tefu, Pten, p38a, rl

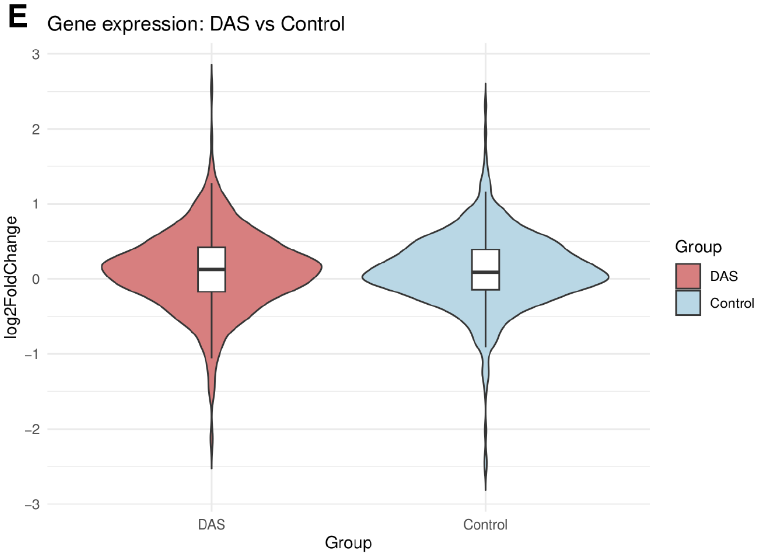

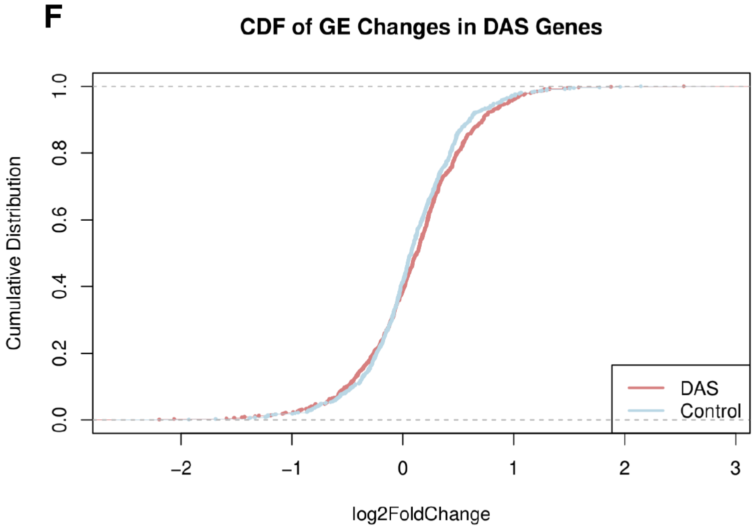

Fig. S3
Even-Ros et al.

Supplementary Figure 3: GO Biological Processes terms of genes expressed differentially or showing AS changes during EC ageing. (A) Workflow undertaken to isolate ECs for GE and AS analyses. Germaria expressing mCD8::GFP under the control of the *ptc-LexA* line in ECs were used to sort cells expressing GFP followed by pico-profiling and RNA-seq. **(B, C)** Representation of the identified terms after a GO-biological processes terms analysis of genes with differential GE (B) and AS (C) changes during EC ageing. NMJ: neuromuscular junction; FGFR: fibroblast growth factor receptor; EFGR: epidermal growth factor receptor. **(D)** List of all genes showing AS changes and that belong to any of the 9 signalling pathways listed in Fig 3e. None of them showed changes in GE. **(E)** Distribution of log2 fold change values of genes with differential AS (DAS) and control genes without. **(F)** Cumulative Distribution Function (CDF) of the genes in the DAS and Control categories. P value of Wilcoxon signed-rank test was >0.05. Related to Figure 3 and Supplementary Data 2.

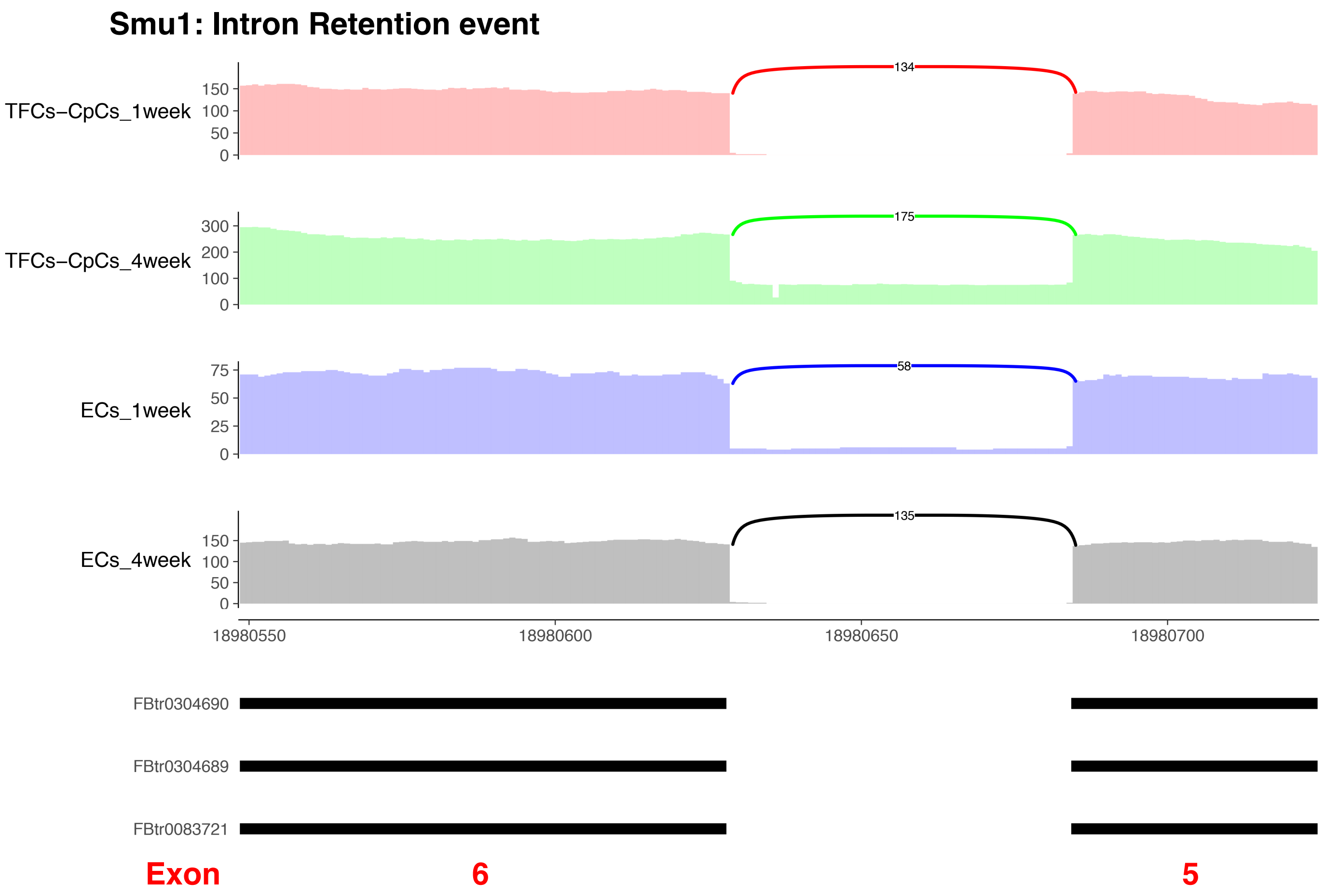

Fig. S4
Even-Ros et al.

Supplementary Figure 4: Sashimi plots of the alternative splicing (intron retention) event in *Smu1* during ageing of TFCs-CpCs and ECs samples. RNA-seq data tracks derived from 1w- and 4w-old samples are shown, with arcs representing splice junctions between exons 5 and 6. X axis refers to genomic coordinates. Y axis refers to number of mapped reads. Numbers across the arcs indicate the number of reads mapped to the junction. Exon numbers correspond to those in Fig. 4. Related to Figure 4.

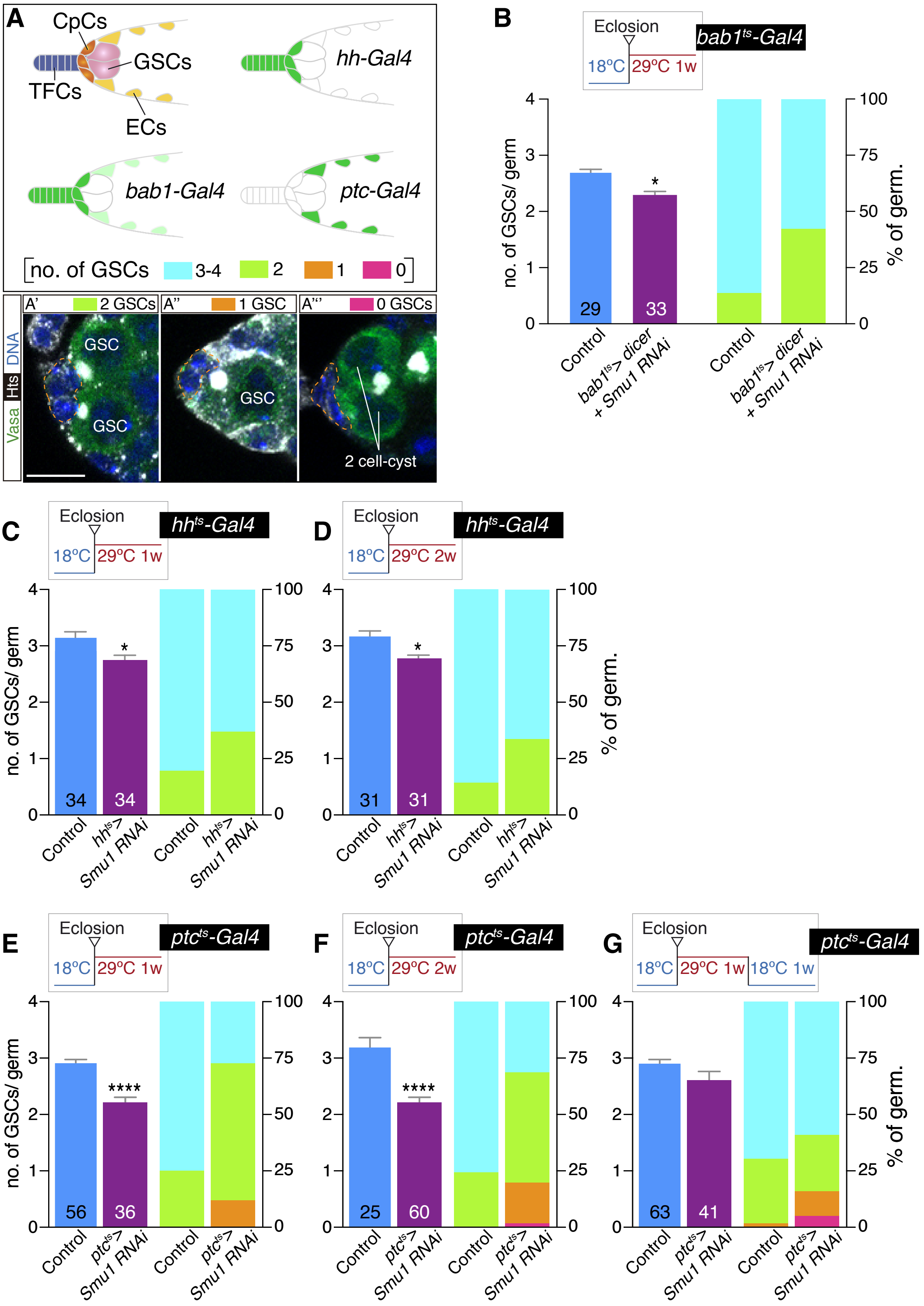

Fig. S5
Even-Ros et al.

Supplementary Figure 5: ***Smu1* is required in niche cells for GSC maintenance. (A)** Scheme of the cell types in which the *bab1*-, *hh*- and *ptc-Gal4* lines are expressed. Colour code for the number of GSCs found in germaria. (A'-A''') Examples of germaria containing 2, 1 or 0 GSCs, respectively. Orange dotted lines outline the CpC cluster. **(B)** Quantification of GSC numbers and percentage of germaria containing 2 or 3-4 GSCs in control and in *bab1^{ts}>Smu1 RNAi + dicer* experimental germaria. Flies were grown at 18°C and, upon eclosion, kept at 29°C for one week prior to dissection. **(C, D)** Quantification of GSC numbers and percentage of germaria containing 2 or 3-4 GSCs in control and in *hh^{ts}>Smu1 RNAi* experimental germaria. Flies were grown at 18°C and, upon eclosion, kept at 29°C for one (C) or two (D) weeks prior to dissection. **(E-G)** Quantification of GSC numbers and percentage of germaria containing 0, 1, 2 or 3-4 GSCs in control and in *ptc^{ts}>Smu1 RNAi* experimental germaria. Flies were grown at 18°C and, upon eclosion, kept at 29°C for one (E) or two (F) weeks prior to dissection. **(G)** Flies were grown at 18°C and, upon eclosion, kept at 29°C for one week and returned to 18°C for one week prior to dissection. Error bars indicate SEM. Numbers in the columns denote the sample size per genotype (n). P values of two-tailed, unpaired t-tests considered statistically significant between different genotypes are shown (*: P≤0.05, ****: P≤0.0001). Scale bar: 10µm. Related to Figure 5.

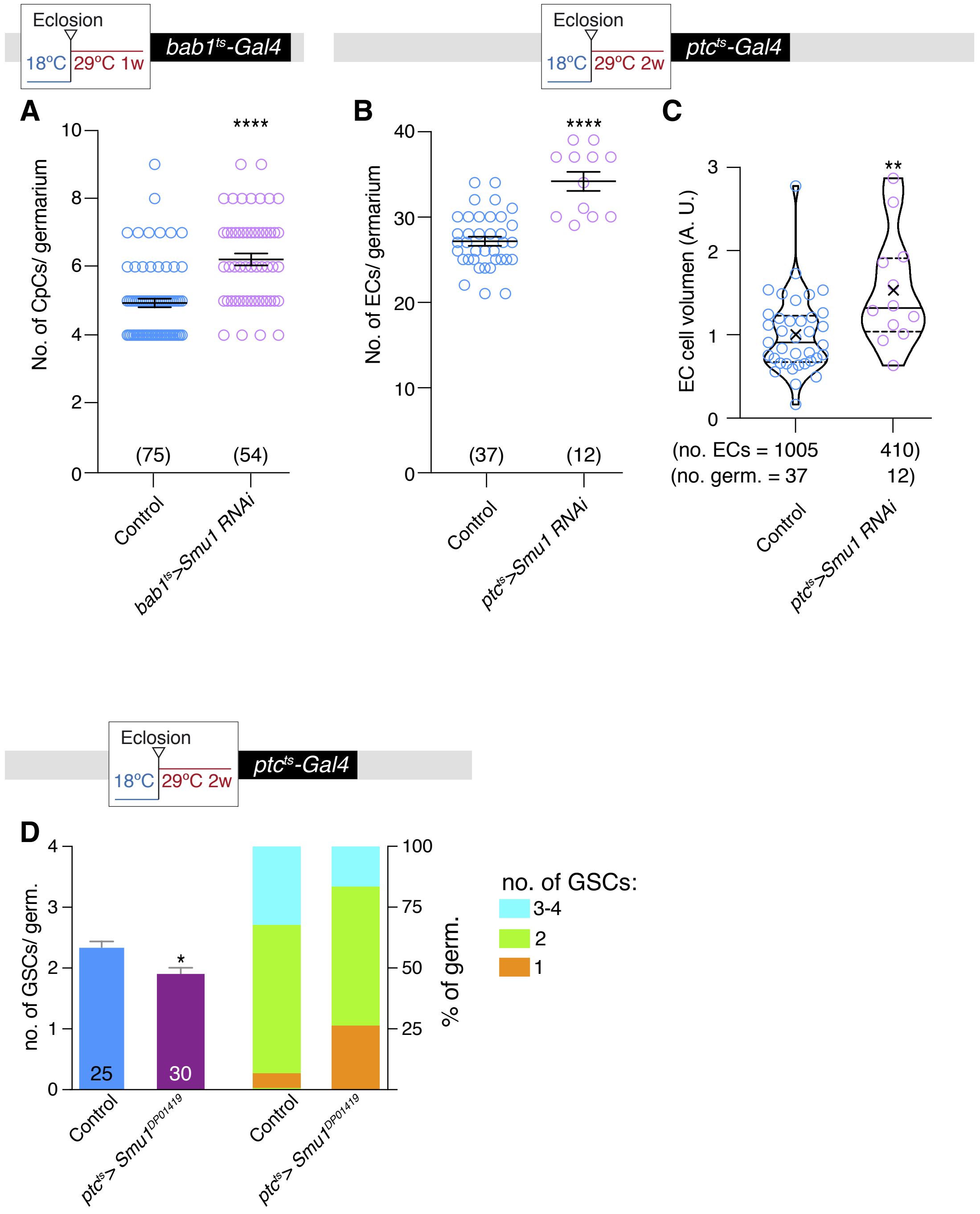

Fig. S6
Even-Ros et al.

Supplementary Figure 6: *Smu1* controls EC proliferation in adult germaria. (A) Number of CpCs per germarium in control and *bab1^{ts}>Smu1 RNAi* experimental germaria grown at 18°C and, upon eclosion, kept at 29°C for one week prior to dissection. **(B)** Number of ECs per germarium in control and *ptc^{ts}>Smu1 RNAi* experimental germaria grown at 18°C and, upon eclosion, kept at 29°C for two weeks prior to dissection. **(C)** The average cell volume of *Smu1 RNAi* ECs is larger than that of control cells. Data correspond to 1005 cells from 37 germaria (controls) and 410 ECs from 12 germaria (experimental). **(D)** Quantification of GSC numbers and percentage of germaria containing 1, 2 or 3-4 GSCs in control and in *ptc1^{ts}>Smu1 DP01419* (*Smu1* overexpression) experimental germaria. Flies were grown at 18°C and, upon eclosion, kept at 29°C for two weeks prior to dissection. Error bars indicate SEM. P values of two-tailed, unpaired t-tests considered statistically significant between different genotypes are shown (*: P≤0.05, **: P≤0.005, ****: P≤0.0001). Related to Figure 5.

A

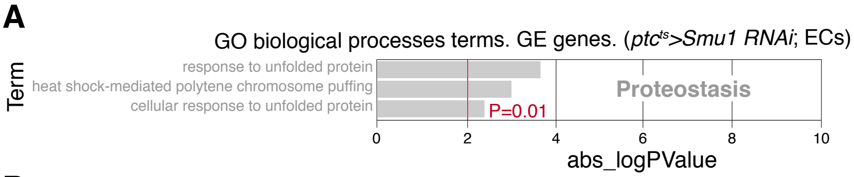

B

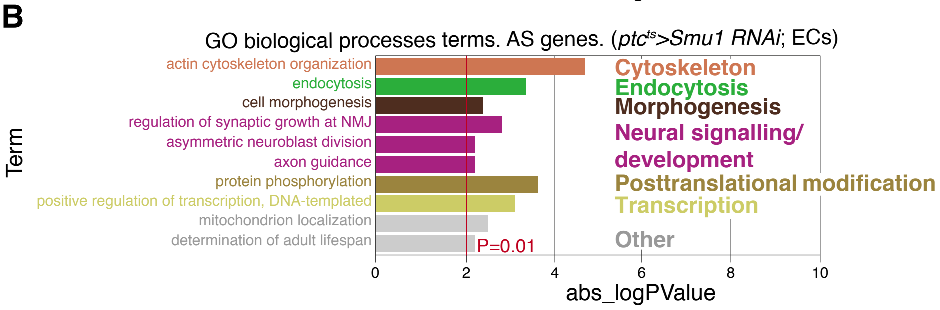

C

KEGG Term	P Value	Genes
Endocytosis	0.010	CenB1A, Eps-15, Efa6, Vps37B, baz, EndoB, Gprk2
Efferocytosis	0.015	CamKII, LRP1, dor, lt, mbc

D

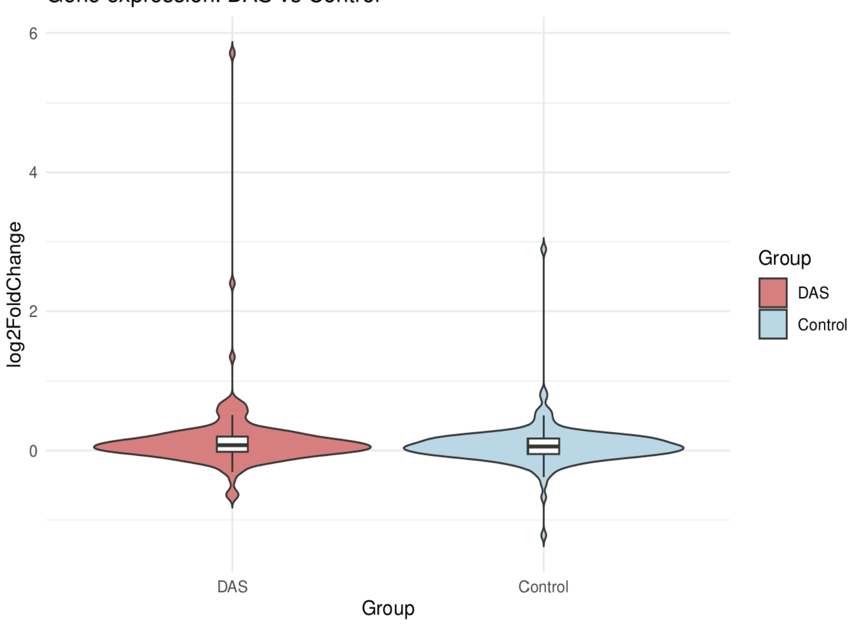

Fig. S7
Even-Ros et al.

E

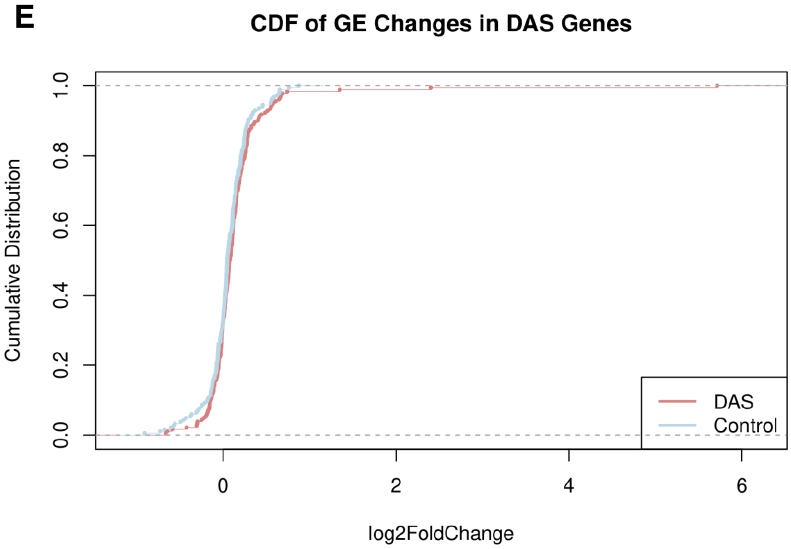

Supplementary Figure 7: GO Biological Processes terms of genes expressed differentially or showing AS changes upon *Smu1* RNAi. (A, B) Representation of the identified terms after a GO-biological processes terms analysis of genes with differential GE (A) and AS (B) changes upon *Smu1* RNAi. NMJ: neuromuscular junction. **(C)** List of all genes showing AS changes and that belong to any of the 2 signalling pathways listed in Fig 6e. None of them showed changes in GE. **(D)** Distribution of log2 fold change values of genes with differential AS (DAS) and control genes without. **(E)** Cumulative Distribution Function (CDF) of the genes in the DAS and Control categories. P value of Wilcoxon signed-rank test was >0.05. Related to Figure 6 and Supplementary Data 6.

Fas2: exon 4

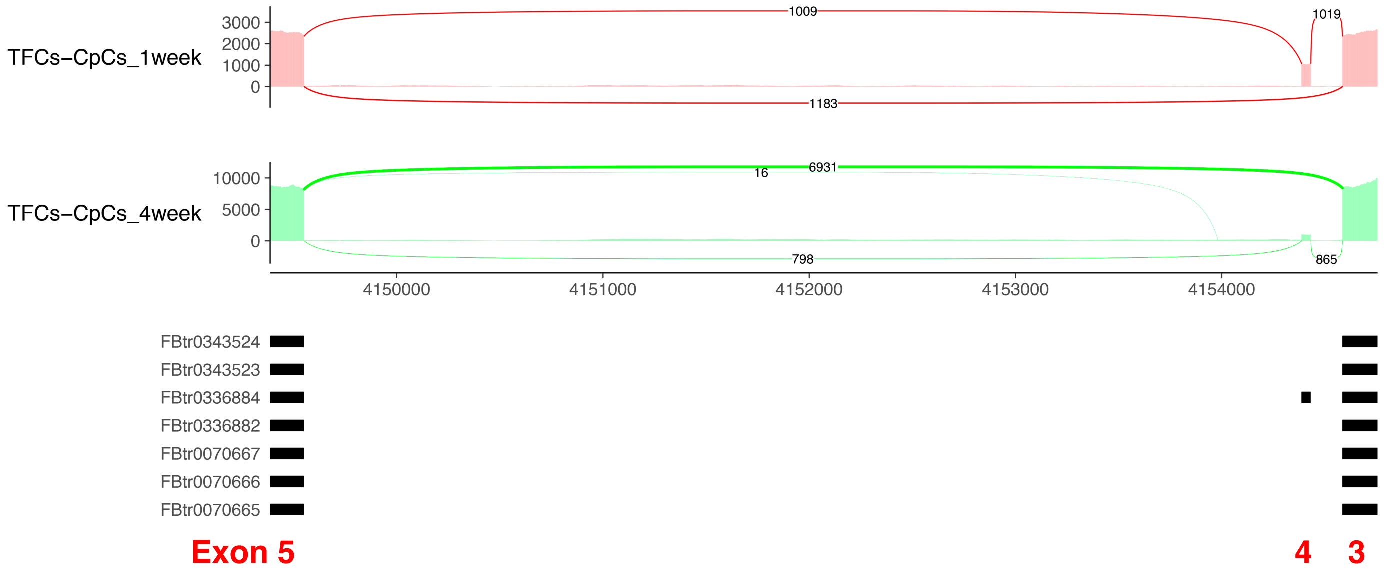

Fas2: exon 12a

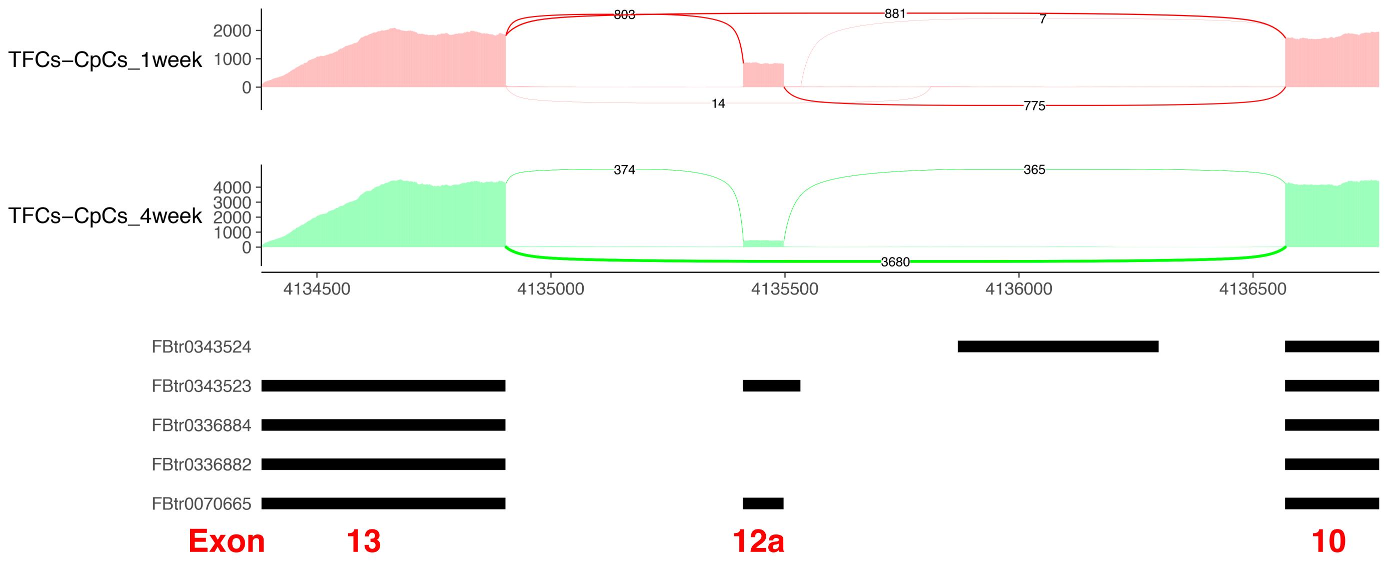

Fig. S8
Even-Ros et al.

Supplementary Figure 8: Sashimi plots of the alternative splicing (exon skipping) events in *Fas2* during ageing of TFCs-CpCs samples. RNA-seq data tracks derived from 1w- and 4w-old samples are shown, with arcs representing splice junctions of exons 3-4-5 or 11-12a-13. X axis refers to genomic coordinates. Y axis refers to number of mapped reads. Numbers across the arcs indicate the number of reads mapped to the junction. Exon numbers correspond to those in Fig. 7. Related to Figure 7.

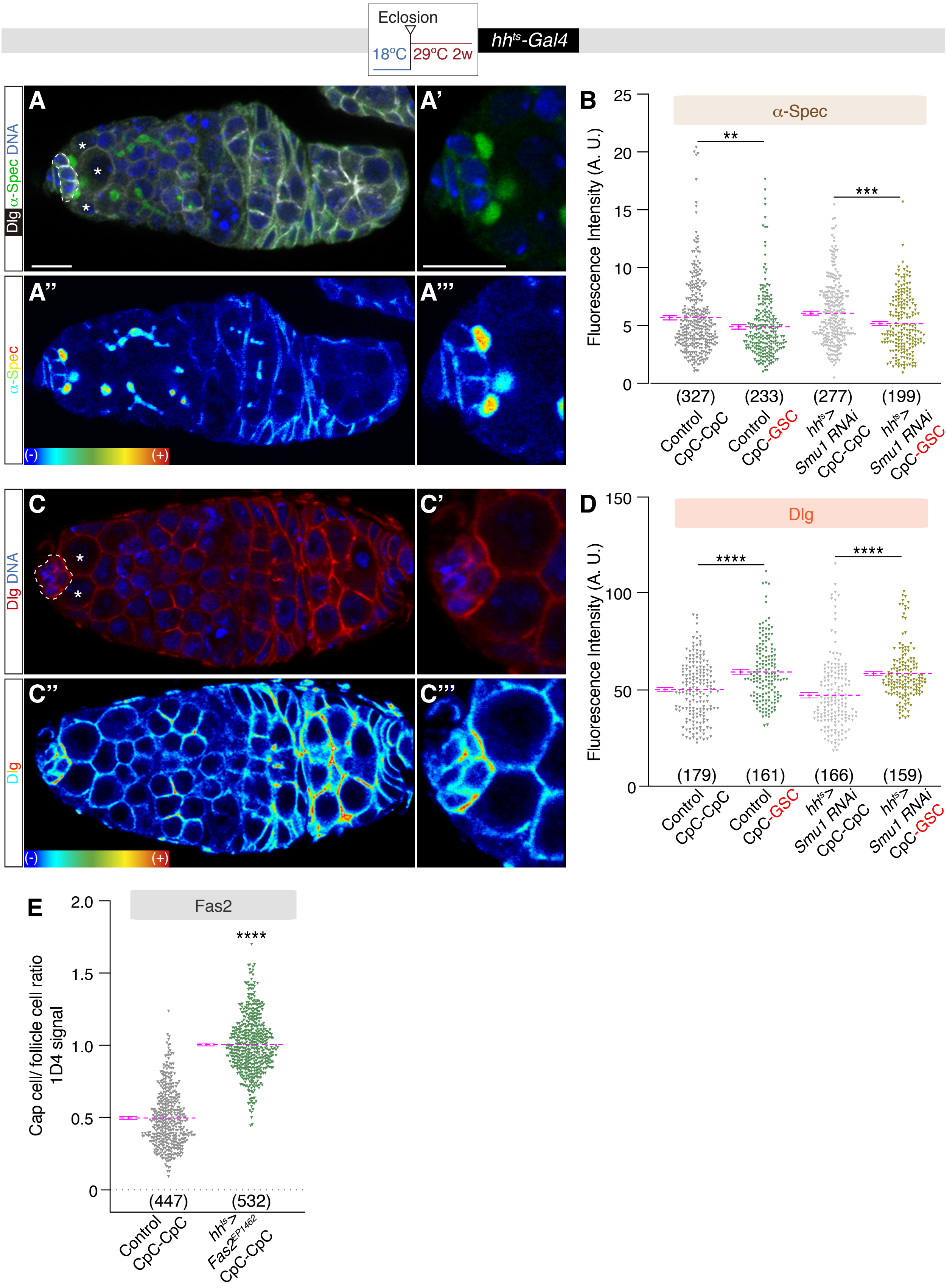

Fig. S9
Even-Ros et al.

Supplementary Figure 9: Polarized distribution of α-Spec and Dlg in CpCs. (A, C) Control germaria showing the distribution of α-Spectrin (A) and Discs large (C). Panels (A’’, A’’’, C’’, C’’’) are false-coloured images representing α-Spec and Dlg signal intensity, respectively. **(B, D)** Quantification of α-Spec (B) and Dlg (D) fluorescence intensity of CpC-CpC and CpC-GSC contacts in control and *hh*^{ts}*>Smu1 RNAi* CpCs (grown at 18°C and placed at 29°C for two weeks upon eclosion). **(E)** Quantification of Fas2 (1D4) fluorescence intensity of CpC-CpC contacts in control and *hh*^{ts}*>Fas2*^{EP1462} CpCs (grown at 18°C and placed at 29°C for two weeks upon eclosion). 1D4 signal is represented as the ratio between the CpC-CpC fluorescent intensity and the average signal from follicle cell membranes from the same germarium. White dotted lines outline CpC clusters. Error bars indicate SEM. Data in (B, D) correspond to 159<measurements<327 obtained from 37<contacts<76 in 16<germaria<38. Data in (E) correspond to 447 (control)<measurements<532 (exp.) obtained from 57 (exp.)<CpC-CpC contacts<69 (control) in 17 (exp.)<germaria<19 (control). P values of two-tailed, unpaired t-tests considered statistically significant between different genotypes are shown (**: P≤0.005, ***: P≤0.0005, ****: P≤0.0001). Asterisks: GSCs. Scale bar: 10µm. Related to Figure 7.
