# Supplementary material for: Drosophila ovarian stem cell niche ageing involves coordinated changes in transcription and alternative splicing
Source: Nat Commun. 2025 Mar 16;16:2596. doi: 10.1038/s41467-025-57901-8 (PMC11911433; doi:10.1038/s41467-025-57901-8)
Supplement: Supplementary file 2 — Description of Additional Supplementary Files [file 41467_2025_57901_MOESM2_ESM.pdf]

# <u>Description of Additional Supplementary Files</u>

**Supplementary Data 1:** List of differentially expressed and differentially spliced genes in 1- week versus 4-week old TFCs + CpCs. Data for Figs. 1, 2 and Supplementary Figs. 1 and 2.

**Supplementary Data 2:** List of differentially expressed and differentially spliced genes in 1- week versus 4-week old ECs. Data for Fig. 3 and Supplementary Fig. 3.

**Supplementary Data 3:** List of differentially expressed and differentially spliced genes in control versus ptcts>Smu1 RNAi ECs. Data for Fig. 6 and Supplementary Fig. 6. Flies were grown at 18oC and placed for two weeks at 29oC upon eclosion.

**Supplementary Data 4:** List of GO terms and KEGG pathways identified in the GSEA conducted with the PANGEA tool. Related to Figs. 1, 3 and 6 and Supplementary Figs. S1, S3 and S6. Selected GO terms had a P value <0.01 and KEGG pathways had a P value<0.1. The selected terms represent the unique terms identified in the corresponding condition pairs (i.e., terms present in 1w and not in 4w lists and vice versa. In the case of the Smu1 RNAi experiment, in control and not in experimental lists and vice versa).
